# Fetor from a Dental Stand-Point

**Published:** 1900-09-15

**Authors:** B. Holly Smith

**Affiliations:** Baltimore, MD.


### FETOR FROM A DENTAL STAND-POINT.

BY B. HOLLY SMITH, BALTIMORE, MD.

Read before the Academy of Stomatology, March 27th, 1900, and reprinted from the *International Dental Journal.*

*Mr. President and Members of the Academy of Stomatology.*—In the domain of professional life dependence of one upon another, which is but faintly foreshadowed in the business world, becomes a fixed and definite principle. The patient, having selected his advisor, as a rule no longer seeks to protect his own interests, except by following the instructions of that advisor. The advisor must be honest, because he is trusted; his attentions must be helpful, because the subject is helpless. The doctor assumes parental authority over his patient, thus becoming a teacher as well as a practitioner; and just in proportion as those practising our specialty appreciate and live up to this high calling will we be regarded as discharging our full obligation to our patients, and taking our place beside other specialists of the great healing art. This contention—

trite and old, if you please, yet bearing the spark of vitality in that it seeks the perpetuation of the life and health of our calling—is my excuse for reciting in your hearing some clinical experiences which, let us hope, will help to establish the thought that the dentist is responsible not alone for the condition of the teeth, but for oral sanitation generally.

The olfactory sense may be regarded as one of the so-called *artistic* senses, and is capable of training and education. People of refinement in all ages have striven to avoid offending it, and not infrequently efforts are made to contribute to the pleasure of the individual through this sense. The delicate perfume of the flower appeals as much to the artistic sense through the olfactories as the coloring of the gracefully moulded petals do through the optics. So well established is this fact that women of refinement vie with each other in the effort to secure a slight though characteristic perfume for their garments, hair, and person. Some have gone so far as to endeavor to associate this perfume with their identity. It is said that whenever Mr. Browning—whose love passes everywhere for ideal—came into the presence of Mrs. Browning he was conscious of the faint odor of violets. And it is intimated that this was a psychic phenomenon produced in the olfactory sense by the extreme mental ecstasy caused by the presence of the adored.

Persons of refinement and culture, of beauty, grace, and good taste, should spread around them everywhere pleasureable impressions. How often do we see these charms negated, and social intercourse become burdensome because of a fetid breath.

To me there is absolutely nothing more beautiful than the human mouth in a state of perfect health, beaded above and below with the most ornamental and precious set of gems, whose beauty is partly concealed by the gracefully curved lips, the pink coloring of which serves to heighten their lustre, the cheeks, the tongue, the palate far back into the pharynx rosy with the tint of health and glistened by the limpid libations of a generous though not too active salivary apparatus, while out and over these comes the expiration of a breath like that of a May morning, having the sweet, fresh, inimitable odor of health.

The treatment of fetor as the subject of a paper may not find favor in the eyes of those who recognize it only as a symptom of some disease and never as a special condition, the amelioration of which requires special care and treatment aside from that prescribed for the many conditions which have it for a symptom. To these let me say that I have selected the subject partly because of the existence of fetor as a special factor in a number of cases of no little interest to me, partly because I think the dentist above any other specialist contributes to the amelioration of this condition, and more than all because I want to establish the contention that the patient must look to the dentist and make him responsible for such relief.

Even though the fetor has nothing to do with the mouth or the area usually included in the dentist's field of work, in the close contact which this specialist sustains to his patient he must be able to recognize its existence and should suggest the propriety of seeking treatment for conditions which the patient seems disposed to neglect. It must be conceded that while other specialists often direct their attentions to the amelioration of conditions giving rise to fetor, the dentist is the specialist most likely to dedect its existence; and it appears to me to be his plain duty to determine its cause; if he can do so, to remove it, if not, he should direct his patient to the specialist best fitted to do so.

Fetor may be said to indicate always a departure from the normal, though its presence is not in every case co-existent with illness of a serious nature. Many persons seem oblivious to it, and medical attention is not always sought even when it is recognized. A mistaken sense of refinement often causes members of a family to conceal their knowledge of its existence from the sufferer, and when the patient becomes aware of it he is ignorant of its cause.

Among the conditions in which fetor plays an important part in determining the presence and progress of disease we may mention bromine, iodine, mercurial, lead, and phosphorus poisonings; caries of the teeth; loose or badly constructed bridges and crown-work; unsanitary plates and appliances; alveolar abscess, pyorrhœa alveolaris; various inflammations, specific and non specific, of the mouth and appendages; fevers, constipation, diarrhœa; in fact, any departure from normal health and tone.

The mouth is lined by pavement epithelium, which is embryologically of the same origin as the epidermis of the skin, and therefore does not belong to the mucous membrane in the same sense as the lining of the stomach, bronchi, etc. There are numerous mucous glands in the mouth which open on the inner surface of cheeks, lips, and on the surface or beneath the tongue. The excretion of these is added to that of the salivary glands to make the complete fluid of the mouth. Many substances, both mineral and alkaloidal, which are absorbed into the circulation are eliminated by these glands as well as the salivary glands, and in being secreted have their influence on the mucous membrane of the mouth. It is a well-known fact that mercury, after it has gotton into the circulation either through absorption by the skin from inunction or by hypodermic injection, is excreted by the mouth, and may give rise to a stomatitis just as severe as if an irritant were applied to the mucous membrane of the mouth.

This is in accordance with the well-known law that substances which are secreted or excreted by mucous membranes act on those mucous membranes.

We notice that these drugs generally cause death to the superficial layers of epithelia, and of course dead epithelium is at once attacked by the saprophytic bacteria of the mouth, notably the staphylococcus pyogenes fœtida.

Miller has shown that these dead epithelia and thickened mucous secretion scraped from a furred tongue show the presence of many rapidly developing organisms. It is only, however, when these organisms act on the dead epithelia and secretions in some little pocket, such as those around the teeth, in the valleculæ of the circumvallate papillæ, or in the crypts in and around the lingual and pharyngeal tonsil, where the air has not free access, that odor occurs.

We find much the same phenomena occuring after the administration of some of the vegetable alkaloids, which are eliminated by the mouth. Atropine checks the secretions of the mouth, and, if pushed, is followed by furred tongue, etc. Other alkaloids also are excreted by the glands of the mouth, and in passing out act on the mucous membrane.

Now, I have no doubt that a similar explanation may be given for the furred tongue and bad breath occurring in many constitutional diseases, such as the fevers, etc. Modern investigation has shown that in the course of these diseases there are developed substances (generally the product of pathogenic organisms) called toxines, which are nitrogen compounds resembling in chemical construction and often in action many of the vegetable alkaloids; the greatest difference is that they are derived from animal instead of vegetable tissues. It seems to me much more logical to refer the furred tongue to the superficial necrosing effect of these toxines during their secretion by the mucous membrane of the mouth than to say it is in sympathy with the other organs of the body, stomach, etc. I do not believe it is a spreading of the condition of the stomach up the long œsophagus to the mouth so much as a simultaneous affecting of both by toxines secreted from the blood by the glands and epithelia of both.

Again, the furred tongue of constipation should be explained in a similar way. It is only one of the many evidences which the body shows of fecal poisoning, making its appearance here much sooner than in other portions of the body. It is not necessary to go into the subject of fecal poisoning to discuss what substances are absorbed from decomposing fæces blocked up in the bowels. Chemistry of the animal products, or physiological chemistry, as it has been termed, which has been developed so much lately since Vaughn extracted his tyrotoxicon from milk and cheese, has shown us that many of the phenomena of disease are to be traced to these products of animal matter,—namely, toxines in living bodies and ptomaines in dead.

Normal fæces contain, besides the residuum of digestion, substances which are excreted, are not intended for use in the economy, but are rather products which are deleterious; such as those in the bile, etc. Now, when the fæces do not pass out in a reasonable time, we have in addition the products of putrifaction, which are even more deleterious. These are absorbed into the blood and carried all over the body, and the mucous membrane of the mouth in eliminating them is affected thereby.

Now, what is the cause of the odor imparted to the breath? These cases of furred tongue in fevers, fecal poisoning, etc. We cannot suppose that such substances may be excreted by the mucous membrane of the mouth without causing a mild degree of stomatitis. Many of the epethelial cells become opaque and lose their vitality, the secretions of the glands are modified, and these conditions afford dead material, which the saprophytes, always present in the mouth develop. Whenever there is a space where these grow and the air does not get free access, as in the valleculæ at the base of the tongue, the pockets between the gums and teeth, or even where the dead epithelia is so thick as to exclude the air from the lower layers, gases may develop and impart an odor to the breath.

Not infrequently the adenoid tissue known as the lingual tonsil is the seat of cheesy deposit or abscess, which gives rise to fetor.

The thick, ropy secretions which are often stagnant behind the folds of the velum palati, between the tonsils, or about the uvula, are often fetid. In the act of sneezing these are brought in the mouth, when the fetor is readily discovered. When the teeth are being cleaned with the brush, if the bristles be pressed far back on the tongue and palate, these secretions will be dislodged by gagging. Patients should be instructed to cleanse the mouth and appendages.

The following are some of the cases, the report of which it is thought might assist in making the application of the attempted teaching of this paper.

Miss M., cashier in a toy-store, had been in bad health for several months; trouble began with what was pronounced la grippe by the homœpathic physician under whose care she remained. After much suffering and intermittent employment, the patient sought dental services for the relief of an aching tooth. On account of pronounced fetor a careful examination was made, suppurating antrum discovered, successfully treated, and patient restored to health.

A number of antrum cases could be mentioned where fetor was the pronounced symptom and where it assisted the diagnosis, but this case is cited because of the fact that but for the fetor the antral suppuration would not have been discovered and prompt relief afforded.

Miss B., who was under my care, was a very handsome and vivacious young woman. I noticed that in spite of the fact that her teeth were in good condition and the mucous membrane had a healthy appearance, her breath had a fetid odor. I called her attention to it so that we might co-operate in finding the cause. A superficial examination of the nasal cavities was made without discovering any cause for the fetor; the throat seemed normal. She was habitually constipated, so means were suggested for the relief of this condition, and she was requested to call in about two weeks. In the mean time I had a visit from her mother requesting that everything be done for the young lady which was possible. Upon her return, she reported improvement in habit, but there was the same close fetid taint to the breath. In a more careful examination of the throat and tongue, I noticed that the circumvallate papillæ on the dorsum lingualis were very prominent; a spoon shaped spatula scraped over this part of the collected mucous of a most fetid character. With a delicate bistoury I cut through one of these papillæ, and found by the side of it an accumulated mass of very fetid matter. Upon consultation with a friend, a throat and nose specialist, we decided to obliterate these fungiform papillæ with electric cautery, which was done with absolute relief of the condition.

A case in the practice of Dr. Cyrus M. Gingrich, of Baltimore: Miss P., a young woman of more than ordinary attractions, cultivated and refined. Members of the family complained that her breath was fetid; the mouth was in apparently healthy condition, teeth not responsible, tongue slightly furred, and mucous scraped from it offensive. A tongue scraper was advised, and the tongue cleansed with a weak solution of carbolic acid; after treatment a radical cure was effected.

A case in the practice of the late Professor Winder;

Mrs. L., a lady of position and wealth, had been under the care of a throat specialist for six months, being treated for catarrh of the Schneiderian membrane, with little improvement. She was obliged on account of extreme fetor to forego social pleasures; conscious of the infirmity, she apologized profusely for having to subject her dentist and friend to the ordeal of attending to her teeth. A careful examination of her mouth revealed a pulpless bicuspid which was slightly tender to percussion. Its extraction was advised and consented to; a diseased antral cavity was found and restored to health; the patient, from being a recluse and invalid, once more resumed her position of prominence in her family and social circle.

Mrs. C., a woman of not very robust constitution, has been under my care for several years. A not very pronounced fetor was discovered at first sitting, and referred to the presence of an unusually large proportion of pulpless teeth; the four incisors and four bicuspids in the upper jaw were in this condition. A small fistula, open and empty, was discoverable near the frænum of the upper lip; looked to be an opening from the right central incisor. No examination of the nasal cavities was made at first sitting, as the patient was regarded as a transient, having only applied for specific operations upon molar teeth, two of which, in the upper jaw, were pulpless; there were also several teeth in this condition in the lower jaw. At a subsequent visit it was learned that the subject was under treatment of a specialist for nasal catarrh. A year after, this patient reported no improvement of special condition, general health not good; invalidism threatened, patient discouraged and gloomy; applied to have filling renewed in right superior second molar; tooth found to be pulpless, but pulp cavity not exposed. Marked fetor was discovered upon opening; then it was found for the first time that some fifteen years previous a great deal of dentistry was done under the influence of a new obtunding agent; and whereas all dentistry previous to this was performed with a great deal of pain, this was done after treatment and without any pain at all. The arsenical "negro in the wood-pile" being suspected, all pulpless teeth were opened, and most of them found to be in fetid condition. A fistula was discovered in the floor of the left nostril. After a month's treatment fetor disappeared, and after a lapse of three years no more catarrhal symptoms have appeared, the patient is restored to health; the upper teeth, which were diseased and unsightly, have been bleached, and the woman is as sweet and handsome as she was in her fifteen-year old form.

The fetor attending fractures is in proportion generally to the comminution, where specula or fragments are broken away; these frequently necrose, and quite a characteristic odor is developed. Added to this a fetor from accumulated *debris* about the splint is to be combated.

A fetid odor is often imparted to the breath from the socket where a tooth has been extracted recently. A partially erupted wisdom tooth about the crown of which the gum is swollen gives a like odor.

I had a patient call for examination whose breath was most fetid. He apologized for it on the ground that his stomach had been out of order. I soon discovered a loose bridge to be the cause of the fetor, but being interested in his diagnosis, I asked him how long he had been suffering. He said that about a month before he had noticed the bad odor when he arose in the morning. He consulted his doctor, who, upon an examination of his tongue, said he would require some purgative medicine, which he took with abundant effect but very little benefit. Since that he had been undergoing a regular course of medicine for indigestion, with not much improvement. He was restored completely by a resetting of the bridge.

On several occasions I have had occasion to warn patients of mercurial salivation, the presence of which I was led to determine by the peculiar fetor associated with this condition.

Your essayist has found permanganate of potash a rock of defence against fetor in all conditions, using it in weak solution, say one grain to the ounce, often having it decolorized by injecting it into suppurating cavities.

### DISCUSSION.

Discussion of Dr. Smith's paper by members of the Philadelphia Academy of Stomatology.

Dr. Brubaker: It seems to me that all the statements that have been made have been based on well-established physiological and pathological facts.

Independent of the local condition of the mouth which has been alluded to, there can be no doubt that much of the disagreeable fetor of the breath is dependent upon the putrefaction of proteid material which occurs in the small intestine. Owing probably to some indiscretions in diet, there is gradually established in the small intestine, more particularly in the neighborhood of the biliary passages, a catarrhal condition which interferes with the proper elimination of bile and probably of pancreatic juice, and, in consequence of this, the proteids undergo various stages of putrefaction, with the development of compounds which are extremely fetid, such as skatol, indol, etc. Some of these compounds, after being absorbed from the intestine, are eliminated under various forms, but there is not much doubt that some of these putrefactive products are absorbed into the blood and eliminated by the pulmonary mucous membrane, and in this way give a very decided odor to the breath. These decompositions which take place in the small intestines are very readily recognizable by well known clinical tests, and can, to a very great extent, be obviated by treatment of this part of the intestinal canal.

In this connection I think it may not be out of place to make a suggestion with reference to the treatment. The treatment of the intestine is very simple, and consists essentially in the removal of this catarrhal state, which is accomplished to a very great extent by the use of pulverized sodium phosphate, given in half-teaspoonful doses with plenty of water daily, especially in the morning. It has an admirable effect, stimulating the intestinal tract and biliary passages. A pill administered at night as required, and consisting of one grain of blue mass, one grain of aloe, and one-quarter of a grain of podophyllin, is one of the best combinations for the removal of those putrefactive changes in the small intestines upon which fetor of the breath very frequently depends. The drinking of an additional quantity of water assists elimination of these odorous emanations. I think that every dentist is privileged to prescribe remedies as simple as those I have mentioned, and I am quite sure that if they are applied this condition of the breath will be largely removed.

Dr. Schamberg: Only recently I had occasion to assist Professor Cryer, at the University, in an operation upon a necrosed maxillary bone, in which there was a considerable amount of hemorrhage and tannic acid was used as the hæmostatic. In this instance we noted a most horrible fetor. It was simply unbearable upon changing the dressing, and was sufficient to make the patient feel temporarily sick. I was quite sure she had been inhaling these obnoxious odors up to the time of the changing of the dressing.

Dr. A. H. Thompson: I should like Dr. Smith to have gone very much more into detail with regard to such antiseptic care of the mouth as would be desirable for us to advise our patients to associate with ordinary hygiene.

We know that accumulations about the teeth give rise to offensive odors, even with persons in the best of health, and that constant care is required to remove particles that may decompose. I think we do not yet have the proper method of what we might call the sanitation of the mouth, nor that we prescribe for our patients what is really ideal hygienic care. Of course, we have some antiseptic washes that can be used, but something more is needed.

Dr. Gaskill: I think attention should be especially directed to the offensiveness of badly constructed bridges and crowns, particularly the so-called "saddle-bridge." Food and foreign substances will crowd beneath it. If we had a perfectly hard surface to deal with it might be different, but the mucous membrane yields and the saddle does not fit. In one case a saddle-bridge had been in the mouth for one year. The saddle was carried back from the cuspid for a distance the width of three teeth on one side, and there was a strip of gold crossing the palate, with a piece of bridge-work on the other. This made a shelf for the deposit of food. When that piece of work was shown to the patient she said, "You could not pay me a thousand dollars to have that put back into my mouth."

Dr. William Trueman: I have listened with a great deal of attention to the essayist, and was particularly impressed by the suggestions which he made and the way in which he broadens the field of dental science. The common idea is to mask fetor of the breath. The next idea is to combat it by some agent that shall neutralize the agent that is producing the bad odor. The Doctor suggests we find and remove the cause, restore the parts to a healthy condition, and then the odor at once ceases.

Dr. Cupid: If we do not understand particularly the cause of the trouble we certainly can not work out its removal or overcome the condition. I think, as has been said by the essayist and other writers previously, that there should be attempted the mechanical removal of this deleterious substance—no doubt waste material — which has been retained in the tissues, particularly in the furred tongue. The dead epithelial layers which should be constantly thrown off are being retained, chemical decomposition is going on, leading up to a profound putrefaction, which necessarily produces intense and persistent odors, and if we can remove them mechanically it is a great step toward overcoming the trouble. If we can go back and remove the cause of the trouble it is still better.

I wish to speak of two different systems that I have followed very carefully and partly successfully. These are the medicinal treatment with permanganate of potassium and the mechanical with the tooth brush. I have found that thorough scraping of the furred tongue and the mucous surfaces has resulted in great improvement, not only in the case of my patients, but with myself, by the removal of collections of dead epithelial tissue and substances that have been retained about the fauces. In every case where I find fetor I invariably prescribe it, together with the use of permanganate of potassium in dilute solution.

Dr. Gaylord: It seems to me that one point has been overlooked in the matter of fetor, and that is the accumulation of calculus about the teeth. It is one of the most important things that come under the eye of the dentist, and which, I fancy, is more or less neglected by the profession, partly owing to its disagreeable nature; but we can not conscientiously dismiss a patient without thoroughly removing this substance. So long as it remains we can not expect a clean, wholesome mouth.

Dr. Rice: The subject of the removal of particles of food that have been retained in the interdental spaces has been dwelt upon, and I am surprised to find that the atomizer has not been spoken of. The mechanical removal of these particles of food is necessary for a lessening of this fetor. I have found that the use of the atomizer is very efficacious, and that the proper projection of the antiseptic upon the tissue is more important than the antiseptic.

Dr. Kirk: I should like to call attention to the point that a fetor of the breath is not always indicative of a pathological condition. In all those conditions where the salivary secretion is suppressed, either temporarily or for a length of time, there is developed, as the essayist has indicated, a tendency to putrefactive changes in the mouth, and this tendency may be developed in a perfectly healthy individual during the night. I think that all who have had the experience of raising children have noticed that even in the mouth of a perfectly healthy infant, where there is no evidence of any caries or other pathological condition, if there is a tendency to mouth-breathing at night, as there may be for a short time, it is quite a common thing for a slight heaviness or disagreeably fetid breath to be developed. It will almost immediately pass away as soon as the salivary secretion is re established. The reason for this would seem to be that the germs of putrefaction do not develop so readily in a mouth in which the saliva is rapidly or normally flowing.

There is another condition which has not been fully dwelt upon, and that is the relation of food habit to fetor of the breath. It is undoubtedly true, as alluded to by Dr. Brubaker, that the digestion of large quantities of proteid food is more apt to produce this odor of the breath than where a vegetable diet is used. I think that may be accounted for by the fact that the activity of the salivary glands is perhaps more stimulated by a vegetable diet than a proteid one, with consequent inhibitory action upon the germs of putrefaction by saliva in quantity.

Dr. B. Holly Smith: I think it has been well said that the importance of oral sanitation, and the means of carrying it out, is a thing that should be dwelt upon. I recall with some chagrin and mortification a patient who came to me some years ago with a mouth in almost perfect condition. The mucous membrane was everywhere of a beautiful, healthy tint. The teeth were white and clean, but at the angles the gum had been brushed away from the cuspids and bicuspids to some extent by what I supposed was a very stiff brush. I counselled the young woman to avoid the use of this stiff brush. She told me then that she used the brush quite a good deal on the soft parts, and her teeth were beautiful and her mouth was in a nice condition. I have never had the courage to say to that young woman what I must say to her when I have a settlement with my conscience, that I did her a great injury by warning her to be careful in the use of her brush. I suggested a softer brush, and now she comes to see me twice a year, and I have calculus to remove and her mouth is not in as healthy a condition as before. There is every evidence of neglect on her part, and I feel that I am a party to it. I now frequently instruct my patients to clean out about the palate and the little circumvallate papillæ, which often grow up like mushrooms. In the case I spoke of, where they were obliterated by the electric cautery, the woman was a charming lady, who occupied a good social position, and after I began to see something of her family and friends they talked about "the great misfortune." Really, it seemed so unfortunate that the young lady had such a bad breath that they did not like to talk to her about it, and it was a source of mortification and embarrassment to all. I had never read anything along this particular line, and I do not know that Dr. Thomas, who assisted me in the operation, had either, but we decided that these little valleys about the fungiform papillæ seemed to fill and retain enough fetid matter to impregnate every breath. Since then I have never had occasion to use the cautery, but I have called the attention of my patients to the use of the broad brush for cleaning the tongue. I have no doubt that the vigorous, though careful, cleaning of the tongue and mucous membrane will aid in the production of a healthy condition.

Dr. Ottolengui removes gold crowns by drilling through the gold crown a little hole about on the line where you would suppose the top of the crown had been attached to the band, on the line of union between the crown and the band, along the buccal surface. Drill that hole into the cement as far as you can conveniently do so. Follow with a round bur, enlarging it without cutting out much of the cement. I get the hole large enough to pass into it a good solid instrument, usually one of the heavy straight pluggers. That is pushed into the hole until it fits snugly; then I have a lever with which to force the crown up. Ordinarily the crown can be tipped off. If it is tight there will probably need to be some up and down movement until it comes of.—*Items of Interest for July.*

